# Enhancing coherent transport in a photonic network using controllable decoherence

**DOI:** 10.1038/ncomms11282

**Published:** 2016-04-15

**Authors:** Devon N. Biggerstaff, René Heilmann, Aidan A. Zecevik, Markus Gräfe, Matthew A. Broome, Alessandro Fedrizzi, Stefan Nolte, Alexander Szameit, Andrew G. White, Ivan Kassal

**Affiliations:** 1 Centre for Engineered Quantum Systems and Centre for Quantum Computation and Communication Technology, School of Mathematics and Physics, The University of Queensland, Brisbane, Queensland 4072, Australia; 2 Institute of Applied Physics, Abbe Center of Photonics, Friedrich-Schiller Universität Jena, Max-Wien-Platz 1, D-07743 Jena, Germany; 3 Institute of Photonics and Quantum Sciences, Heriot-Watt University, Edinburgh EH14 4AS, UK

## Abstract

Transport phenomena on a quantum scale appear in a variety of systems, ranging from photosynthetic complexes to engineered quantum devices. It has been predicted that the efficiency of coherent transport can be enhanced through dynamic interaction between the system and a noisy environment. We report an experimental simulation of environment-assisted coherent transport, using an engineered network of laser-written waveguides, with relative energies and inter-waveguide couplings tailored to yield the desired Hamiltonian. Controllable-strength decoherence is simulated by broadening the bandwidth of the input illumination, yielding a significant increase in transport efficiency relative to the narrowband case. We show integrated optics to be suitable for simulating specific target Hamiltonians as well as open quantum systems with controllable loss and decoherence.

## Abstract (web summary)

The efficiency of coherent transport can be enhanced through interaction between the system and a noisy environment. Here, Biggerstaff *et al*. report an experimental simulation of environment assisted coherent transport using laser-written waveguides, showing that controllable decoherence yields an increase in transport efficiency.

## Graphical abstract

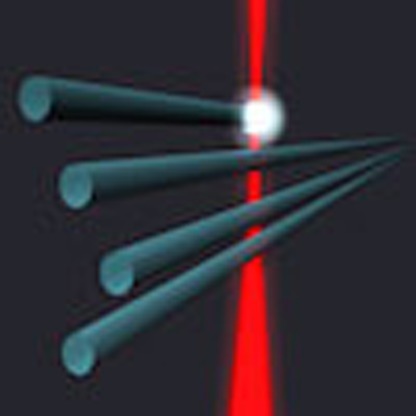

Recent research into photosynthetic antenna complexes has shown evidence of coherence in excitonic energy transport^1^,^2^,^3^,^4^, despite the noisy cellular environment in which such complexes are found. Indeed, environmental decoherence has been credited with increasing the efficiency of transport through these systems, an effect known as environment-assisted quantum transport (ENAQT)^5^ or dephasing-assisted transport^6^,^7^. While ENAQT has been the subject of many theoretical studies—whether in the photosynthetic context^8^,^9^,^10^,^11^,^12^,^13^,^14^,^15^,^16^ or in other nanoscale transport systems^17^,^18^,^19^,^20^—and despite its potential for improving transport in artificial quantum systems, it has so far never been directly observed.

ENAQT can be understood by considering a single excitation on a network of *N* coupled sites, governed by a tight-binding Hamiltonian^5^


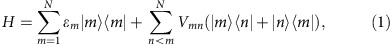


where 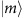 denotes the excitation being localized at site *m*, *ɛ*_*m*_ the energy of that site and *V*_*mn*_ the coupling between sites *m* and *n*. Although ENAQT can occur on an ordered lattice where all the energies *ɛ*_*m*_ are equal^21^, transport enhancement was first explained in disordered systems, which we consider here.

In most studies, ENAQT is about the efficiency of transport from a particular initial site to a particular target site, where the excitation is irreversibly trapped. In the case of a photosynthetic complex (Fig. 1a), trapping describes the transfer of excitons to a reaction center, where they drive charge separation. It can be modelled as irreversible coupling of the target site to a sink at rate *κ*. The efficiency is usually defined as the probability of finding the exciton in the sink after a certain time or, more commonly, in the long-time limit.

ENAQT occurs when adding decoherence increases the trapping probability above the fully coherent case. In general, decoherence results from coupling of a quantum system to inaccessible degrees of freedom. For example, in photosynthetic antenna complexes, the energies of chromophores are coupled to molecular vibrations; tracing out this environment results in decoherence in the excitonic subspace.

ENAQT can be understood in several different ways: as suppression of destructive interferences, as bringing of neighbouring sites into resonance, or as transitions between otherwise-stationary eigenstates. The first of these interpretations starts from the fact that energetic disorder tends to localize the wavepacket through processes such as destructive interference or Anderson localization^22^,^23^,^24^, thus preventing it from reaching the target. Adding decoherence diminishes these coherent processes, increasing the transport efficiency to the target. The second view is that decoherence—if it is in the site basis, as is often assumed—can be modelled as fluctuations of site energies. On that view, increasing fluctuations enhance site-to-site transfer by bringing neighbouring sites into transient resonance more frequently. The third approach is to note that the eigenstates of *H* are stationary in the absence of decoherence, making it difficult to reach the target if the initial and target sites differ in energy. Incoherent processes, however, permit transitions between the eigenstates of *H*, yielding greater mobility. Finally, in some specialized cases ENAQT can also be understood as an instance of a phonon antenna^25^ or of momentum rejuvenation^26^.

The earliest theoretical studies of ENAQT focused on the case where the decoherence takes the form of site-independent, Markovian, pure dephasing^5^,^6^. However, any form of decoherence, including non-Markovian ones^27^,^28^, can increase transport efficiency as long as it allows population transfer between otherwise-stationary eigenstates.

Here we use an integrated photonic simulator to demonstrate the first implementation of ENAQT. Our simulator was fabricated using femtosecond-laser direct writing, which allows waveguides to be drawn directly into glass using a focused, pulsed laser. This permits the creation of three-dimensional waveguide arrays, as well as precision and repeatability in engineering interactions^29^,^30^,^31^,^32^,^33^,^34^,^35^,^36^. We use control over the wavelength and bandwidth of the guided light to simulate effective decoherence, thereby enhancing transport efficiency.

## Results

### Theoretical model

We simulate ENAQT in an array of coupled single-mode optical waveguides obeying the equation^37^,^38^


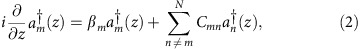


where the light is propagating in the *z* direction, 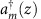 is a creation operator for a photon in waveguide *m* at position *z*, and *β*_*m*_ and *C*_*mn*_ are the propagation constants of the waveguides and the couplings between them, respectively. The former are determined by the waveguides' refractive index profiles, while the latter also depend on the separations between them. Light propagation governed by this Schrödinger-like equation directly simulates evolution under *H*, with *C*_*mn*_ replacing *V*_*mn*_ and the *β*_*m*_ replacing *ɛ*_*m*_. We can thus simulate different Hamiltonians by controlling the number, position and refractive indices of the waveguides.

The intrinsic stability of laser-written waveguide arrays renders decoherence challenging to simulate. One approach is to stochastically modulate the index of refraction along each waveguide^39^. Although every realization with a particular longitudinal index profile will be fully coherent, decoherence can be simulated by averaging over the recorded optical outputs from many realizations in post-processing. This approach was recently used to simulate decoherence-enhanced navigation of a maze^40^.

By contrast, we simulate decoherence by averaging over the results from a single array illuminated with many optical wavelengths. Although each individual wavelength propagates through the waveguide array coherently, effective decoherence can be achieved using broadband illumination and a single output intensity measurement that does not resolve wavelength. In other words, the wavelength degree of freedom is traced out yielding a partially mixed state. Similar approaches are well established in optics, where, for instance, thick birefringent quartz plates followed by wavelength-insensitive measurements have been used to decohere the polarization states of single photons^41^,^42^.

As an example of our approach to simulating decoherence, we consider two uncoupled waveguides *a* and *b* that have a difference Δ*β* in their propagation constants at a wavelength *λ*_0_. As light propagates along the waveguides, it will accumulate a phase difference *z*Δ*β* between them. Broadband illumination can then be seen to cause effective decoherence on a length scale comparable to the illumination coherence length. Any initial coherence *ρ*_*ab*_ between the waveguides will decay as


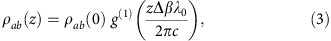


where *g*^(1)^(*t*) is the normalized first-order temporal correlation function of the light, which—being proportional to the Fourier transform of the spectrum—decays to zero faster for spectrally broader illumination. Because Markovian dephasing causes exponential decays of coherences, the dephasing in equation (3) is non-Markovian unless the light has a Lorentzian spectrum.

The overall strength *γ* of the decoherence can be quantified as the inverse of the optical coherence length, 
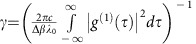
, and is usually proportional to the full-width at half-maximum bandwidth Δ*λ*. For a uniform distribution centred at *λ*_0_,


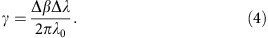


In the case of coupled waveguides, wavelength dependence affects couplings in addition to the propagation constants. The resulting decoherence will therefore not only have characteristics of pure dephasing (as in equation (3)), but will also include off-diagonal terms. However, this kind of decoherence can also result in ENAQT. Indeed, the off-diagonal terms imply that our decoherence could, in principle, be used to simulate ENAQT in ordered systems^21^, but the effect would be weaker because of the absence of the pure-dephasing contribution when Δ*β*=0.

### Experimental results

**Figure 1:**
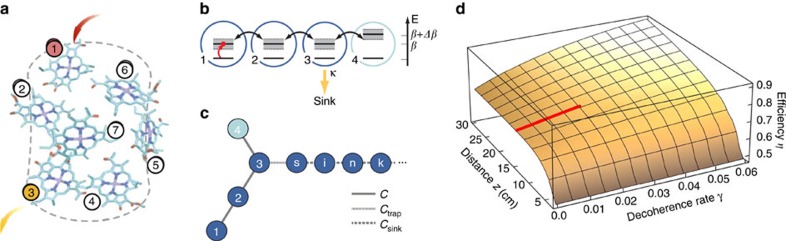
Environment-assisted quantum transport (ENAQT). (**a**) Photosynthetic antenna complexes are networks of chlorophylls that collect and transfer solar energy. A well-studied example is the Fenna–Matthews–Olson complex of green sulphur bacteria, here depicted as a network of seven sites that transports excitation energy from initial site 1 to target site 3 (adapted with permission from ref. 49). Simulations have suggested that this transport may be enhanced by decoherence^5^,^6^,^7^. (**b**) We simulate an instance of ENAQT on a lattice of four sites, with site 1 initially excited and site 3 the target. If the detuning Δ*β* of site 4 equals *C*, one of the system eigenmodes has no occupancy at site 3 and cannot couple to the sink; by broadening the levels, decoherence breaks the condition Δ*β*=*C*, allowing all eigenmodes to couple to the sink and thus increasing transport efficiency. (**c**) Our simulator consists of four coupled waveguides arranged as shown (cross-section). The sink is modelled with a large array of closely coupled waveguides that transport light away from the main four waveguides. At the central wavelength *λ*_0_, waveguide 4 has propagation constant *β*+Δ*β*, while the others have propagation constant *β*. (**d**) Theoretical expectation of transport enhancement for this system, as a function of simulation length *z* and decoherence strength *γ*. The red bar indicates the region explored experimentally.

For our simulation, we chose the network of four sites shown in Fig. 1b because it is one of the smallest systems in which ENAQT is possible and because it can give significant enhancements even with relatively weak decoherence. We simulated this network using waveguides arranged as in Fig. 1c, where waveguide 1 was the input, waveguide 3 the target and the sink consisted of a long linear array of tightly coupled waveguides. The coupling between waveguides in this sink was significantly higher than between the four main waveguides, so that any light entering the sink from waveguide 3 was largely transported away^43^,^44^,^45^. The sink need only be long enough to prevent light reflected from the far end from returning into the main simulation waveguides.

We chose propagation constants and separations among the four main waveguides in order to best approximate the Hamiltonian corresponding to Fig. 1b,


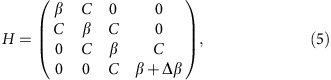


where all parameters depend on the wavelength *λ* of the input light. Due to their wide separations, couplings between non-neighbouring sites are negligible (<5% of neighbouring-site couplings). Our simulator is designed so that, at our central simulation wavelength *λ*_0_,


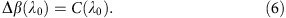


In this case, one of the eigenstates of *H* has no support on site 3, 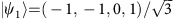, while the remaining three eigenstates all have substantial support on site 3. Because 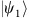 cannot couple to the sink—at least at *λ*_0_—the maximum trapping efficiency at infinite time is 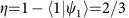.

Considering the wavelength dependence of *H* provides a different way to see the decohering effects of broadband illumination. Due to the dependence of *β* and *C* on the wavelength, equation (6) only holds at a particular wavelength *λ*_0_. At other wavelengths, 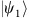 will have some support on waveguide 3 and thus be able to couple to the sink, increasing the efficiency above 2/3, as shown in Fig. 1d. Unlike in other examples of ENAQT^5^, the efficiency increases monotonically with the strength of decoherence, meaning there is no optimal level of decoherence in this model.

**Figure 2:**
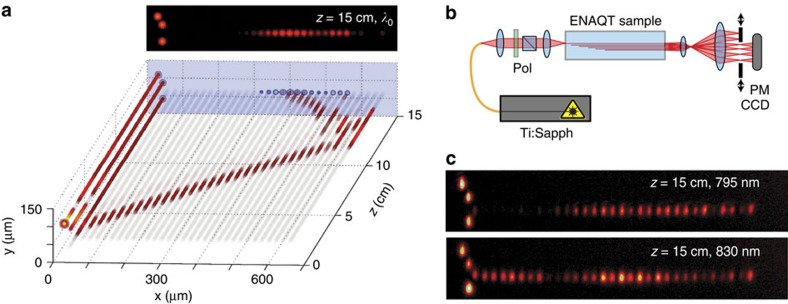
Experimental setup and waveguide design. (**a**) Predicted dynamics of light in our waveguide array, as a function of *z*. The inset shows the predicted device output distribution for input at wavelength *λ*_0_. The sink array is sufficiently long that light reflecting from the far boundary fails to couple back into the system waveguides during the simulation. (**b**) A fibre-coupled, tunable Ti:sapphire laser in quasi-cw mode undergoes polarization control (Pol) before it is imaged into the sample using a 15 mm focal-length aspheric lens. The output is imaged via a 14 × telescope onto a variable slit, which collectively measures the total intensity output from the system, bath or all the waveguides using a large-area power-meter (PM). Alternatively, the output can be imaged onto a CCD camera for alignment and diagnostics. (**c**) CCD images of the output after 15 cm when illuminated at *λ*_0_=792.5 nm and at 830 nm. As designed, the light in the system waveguides is evenly distributed at *λ*_0_ apart from the target site, which is dark. In contrast, the target site is much brighter at 830 nm, indicating that more light will couple into the sink—a sign of ENAQT.

On the basis of measurements of couplings and propagation constants in isolated pairs of waveguides (see Methods xsection), we selected the following design parameters: Δ*β*=*C*=1.0 cm^−1^, *C*_trap_=1.5 cm^−1^, and *C*_sink_=1.75 cm^−1^ (Fig. 1c). Figure 2a shows numerical modelling of light propagation given these parameters. Although these were designed for a center wavelength of 800 nm, variations in the implementation of the waveguide parameters resulted in equation 6 being satisfied at *λ*_0_=792.5 nm.

**Figure 3:**
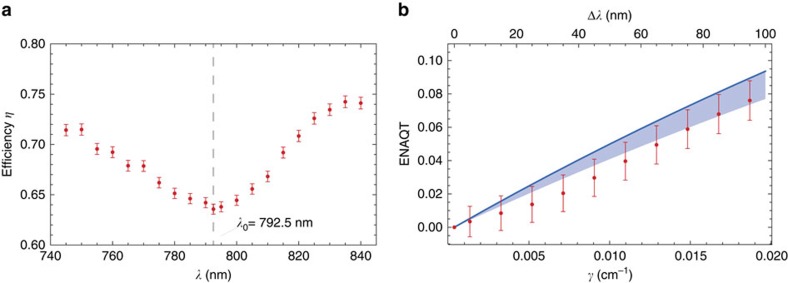
Magnitude of observed ENAQT. (**a**) Transport efficiency—the portion of light that makes it to the sink—as a function of wavelength. The minimum efficiency at *λ*_0_=792.5 nm is *η*=0.636±0.002, slightly less than the theoretical infinite time limit of 2/3. The error bars are s.d. caused by imperfect repeatability in coupling light into the sample and laser power fluctuations. (**b**) ENAQT—the relative increase in the efficiency over that at *λ*_0_—as a function of the optical bandwidth (top horizontal axis) and corresponding decoherence strength *γ* (bottom horizontal axis). The red points are obtained by averaging the measured efficiencies over a uniform broadband spectrum with width Δ*λ* and centred at *λ*_0_. The blue line represents the theoretical ENAQT, calculated based on the model in Fig. 1b and containing no free parameters (it is the cross-section of Fig. 1d along the red line segment). The shaded region represents possible ENAQT if Δ*β*(*λ*_0_) deviates from *C*(*λ*_0_) by up to 10%.

Our experimental setup is shown in Fig. 2b. We measured the efficiency using narrowband light (less than 1 nm bandwidth and always horizontally polarized for consistency) from a tunable Ti:sapphire laser (Spectra-Physics Tsunami) in quasi-cw mode (Fig. 2b). The output was imaged using a custom-built 14 × magnifying telescope and the optical power was measured using a large-area power-meter after isolating either the system or sink waveguides using a variable slit. Examples of the output distribution are given in Fig. 2c, showing the significant difference between illumination at *λ*_0_ and an off-centre wavelength. Figure 3a shows the measured efficiency (fraction of light output in the sink modes) for wavelengths ranging from 745 to 835 nm.

ENAQT—shown in Fig. 3b—is the average enhancement in efficiency over the spectral band of interest, relative to the efficiency at *λ*_0_,


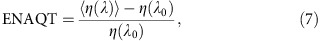


where the average 〈⋯〉 over *λ* is taken over the top-hat distribution on 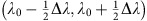. As predicted, it is an increasing function of the bandwidth, that is, of the decoherence strength *γ*. The highest ENAQT observed was (7.6±1.2)% at a bandwidth of 95 nm. We thus demonstrated that coherent transport in a coupled, statically disordered system can be enhanced through decoherence.

## Discussion

The theoretical prediction in Fig. 3b contains no free parameters. It is a simulation of the dynamics under the simulated Hamiltonian *H*, together with trapping from site 3 at the rate *κ*, discussed in Methods section, and pure dephasing between waveguide 4 and the other three waveguides at the rate *γ*. The disagreement between theory and experiment is small considering the number of possible contributing factors. These include the off-diagonal decoherence when the waveguides are coupled, the fact that the trapping is not perfectly exponential, errors in the measurements of the coupling constants, optical losses and error in satisfying equation 6. As an example, the shaded band in Fig. 3b represents the error that would arise if Δ*β*(*λ*_0_) deviated from *C*(*λ*_0_) by up to 10%.

In our experiment, the magnitude of ENAQT was limited by the maximum achievable decoherence, which at *γ*=0.02 cm^−1^ was small compared with the inverse of the propagation length. We were limited by two components of equation (4): the tunability of our laser limited Δ*λ*, while Δ*β* was limited (via equation (6)) by the need to keep *C* small enough to stay in the tight-binding approximation. These are not fundamental limitations, and future improvements that increase *γ* would result in significantly larger transport enhancement; as shown in Fig. 1d, the transport efficiency of this model can get arbitrarily close to 1 for sufficiently long propagation distances and decoherence strengths. Stronger decoherence would also allow ENAQT to be observed in networks that are less sensitive to decoherence than our model.

Our results demonstrate that integrated photonics is well-suited for simulating open quantum systems and capable of implementing a disordered target Hamiltonian with controllable loss. Using broadband excitation to introduce tunable levels of decoherence is the first technique to simulate an open-quantum system in a photonic architecture; previously, low intrinsic noise in photonic devices rendered decoherence difficult to realize in integrated optics, particularly without averaging over results from many different device realizations. Future work will determine the full range of decoherence processes that can be simulated in this way (for example, by modifying the illumination spectrum) as well as ways to extend it to enable the simulation of a greater range of open-quantum systems. Controllable decoherence will not only enhance photonic simulation, but will also permit photonic implementations of quantum-computational methods that take advantage of decoherence^46^,^47^,^48^. In addition, analogies with and extensions of our approach will aid in the experimental optimization of transport in other engineered quantum systems.

## Methods

### Waveguide fabrication

The waveguides were fabricated in high-purity fused silica (Corning 7980) using a laser direct-write technique whereby Ti:sapphire laser pulses are tightly focused into the sample, which is then translated in three dimensions to yield continuous regions of positive refractive index change, which act as waveguides^30^. To obtain single-mode waveguides with the desired propagation and coupling characteristics, laser pulses of duration 150 fs, energy 400 μJ, central wavelength 800 nm and repetition rate 100 kHz were focused 400 μm below the surface using a × 40 microscope objective and translated at 75 mm min^−1^ (for waveguides other than 4).

Permanent index changes result in the focus, yielding elliptical, vertically oriented, single-mode waveguides with modes ∼17 × 19 μm in size at 800 nm. Waveguide 3 and the sink waveguides are in a plane parallel to the surface, while angles of 120° between this plane and the other system waveguides minimize non-neighbour coupling. Because couplings between elliptical waveguides are dependent on angular orientation^29^, we determined the couplings by writing pairs of waveguides oriented at the specified angles. Couplings as a function of separation were determined by writing the pairs at different separations and measuring the output intensities after a known propagation length when only one is optically excited.

The final waveguide separations were chosen to ensure that the tight-binding approximation is maintained, that *C* can be matched by Δ*β*, that *C*_sink_>*C*_trap_>*C* and that the number of sink modes remains manageable. The system-sink population transfer can be modelled as a constant effective rate^43^ 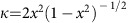 if 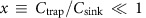. In our case, although *x*=0.86 meant that the decay was not perfectly exponential, it was nevertheless effectively irreversible, considering that sufficiently many sink modes were present to prevent reflection from the far end and back into the main waveguides.

For waveguide 4, the writing translation speed was decreased in order to increase the propagation constant. In a pair of waveguides with mismatched propagation constants, the maximum power transfer depends only on the ratio Δ*β*/*C*. We used this fact to determine the translation speed decrease necessary to set Δ*β*=*C* at 800 nm. Due to the measurement uncertainty (about 8%) and day-to-day variability in the waveguide writing process, several waveguide arrays were written with slightly different writing speeds for waveguide 4 to ensure that one set would yield Δ*β*=*C* in our desired wavelength range. In the best sample, the writing speed was decreased by 12%.

## Additional information

**How to cite this article:** Biggerstaff, D. N. *et al*. Enhancing coherent transport in a photonic network using controllable decoherence. *Nat. Commun.* 7:11282 doi: 10.1038/ncomms11282 (2016).
